# Dietary iron restriction alleviates renal tubulointerstitial injury induced by protein overload in mice

**DOI:** 10.1038/s41598-017-11089-0

**Published:** 2017-09-06

**Authors:** Yasumasa Ikeda, Yuya Horinouchi, Hirofumi Hamano, Tasuku Hirayama, Seiji Kishi, Yuki Izawa-Ishizawa, Masaki Imanishi, Yoshito Zamami, Kenshi Takechi, Licht Miyamoto, Keisuke Ishizawa, Ken-ichi Aihara, Hideko Nagasawa, Koichiro Tsuchiya, Toshiaki Tamaki

**Affiliations:** 1 Department of Pharmacology, Institute of Biomedical Sciences, Tokushima University Graduate School, Tokushima, Japan; 2 Department of Pharmacy, Tokushima University Hospital, Tokushima, Japan; 3 Laboratory of Pharmaceutical and Medicinal Chemistry, Gifu Pharmaceutical University, Gifu, Japan; 4 Department of Nephrology, Institute of Biomedical Sciences, Tokushima University Graduate School, Tokushima, Japan; 5 Department of Clinical Pharmacology and Therapeutics, Institute of Biomedical Sciences, Tokushima University Graduate School, Tokushima, Japan; 6 Clinical Trial Center for Developmental Therapeutics, Tokushima University Hospital, Tokushima, Japan; 7 Department of Medical Pharmacology, Institute of Biomedical Sciences, Tokushima University Graduate School, Tokushima, Japan; 8 Department of Community Medicine for Diabetes and Metabolic Disorders, Institute of Biomedical Sciences, Tokushima University Graduate School, Tokushima, Japan

## Abstract

Increased proteinuria causes tubulointerstitial injury due to inflammation in chronic kidney disease (CKD). Iron restriction exhibits protective effects against renal dysfunction; however, its effects against protein overload-induced tubulointerstitial damage remain unclear. Here, we investigated dietary iron restriction effect on tubulointerstitial damage in mice with protein-overload tubulointerstitial injury. Renal tubulointerstitial injury in animal model was induced by intraperitoneal injection of an overdose of bovine serum albumin (BSA). We divided mice into three groups: normal saline + normal diet (ND), BSA + ND, and BSA + iron-restricted diet (IRD). BSA overload induced renal tubulointerstitial injury in the ND mice, which was ameliorated in the IRD mice. Inflammatory cytokines and extracellular matrix mRNA expression was upregulated in BSA + ND mice kidneys and was inhibited by IRD. BSA-induced increase in renal superoxide production, NADPH oxidase activity, and p22^phox^ expression was diminished in the IRD mice. IRD suppression increased BSA-induced renal macrophage infiltration. Moreover, BSA mice exhibited nucleotide-binding oligomerisation domain-like receptor pyrin domain-containing protein (NLRP) inflammasome activation, which was inhibited by IRD. Ferrous iron increased in kidneys with BSA overload and was inhibited by IRD. Thus, iron restriction inhibited oxidative stress and inflammatory changes, contributing to the protective effect against BSA overload-induced tubulointerstitial injury.

## Introduction

The number of patients with chronic kidney disease (CKD) has been increasing worldwide, and the presence of CKD worsens morbidity and mortality. Proteinuria, including albuminuria, is a biomarker for CKD, is associated with renal tubular and tubulointerstitial damage, and causes further progression of kidney injury and deterioration of renal function^1, 2^. Proteinuria induces tubulointerstitial injury through apoptosis^3^, inflammation^4^, epithelial–mesenchymal transition^5^, and oxidative stress^6^. Recent studies have demonstrated that nucleotide-binding oligomerisation domain-like receptor pyrin domain-containing protein 3 (NLRP3) inflammasome also plays a crucial role in the progression of tubulointerstitial damage induced by protein overload^7, 8^. Thus, proteinuria is involved in further exacerbation of CKD through various contributing factors.

Iron is an essential trace metal for metabolic processes and enzyme activity in all living organisms. Moreover, iron catalyses the Fenton reaction and consequently causes oxidative stress *via* hydroxyl radical production^9^. Therefore, intracellular iron is restrictively controlled by iron transporters, iron-binding proteins, and iron-storage proteins^10^. Of late, it has been demonstrated that the kidney participates in the regulation of iron metabolism and homeostasis^11^; therefore, iron is potentially involved in renal pathophysiology. In different experimental animal models, iron is involved in determining the pathological condition of CKD such as diabetic nephropathy^12–14^, hypertensive kidney injury^15, 16^, and renal fibrosis^17^, and these diseases are ameliorated by iron reduction in the body. Dietary iron restriction inhibits the increment of urinary albuminuria in *db/db* mice^12^; however, it remains unclear whether iron restriction can prevent protein overload-induced renal injury.

In the present study, we determined that dietary iron restriction alleviates protein overload-induced tubulointerstitial injury, and inhibits oxidative stress and inflammatory changes in mice. Moreover, ferrous iron production is elevated in the kidneys with protein overload, suggesting its involvement in the pathological condition of protein-overload renal injury through oxidative stress *via* the Fenton reaction.

## Results

### Effects of dietary iron restriction on BSA-induced tubulointerstitial injury

**Figure 1:**
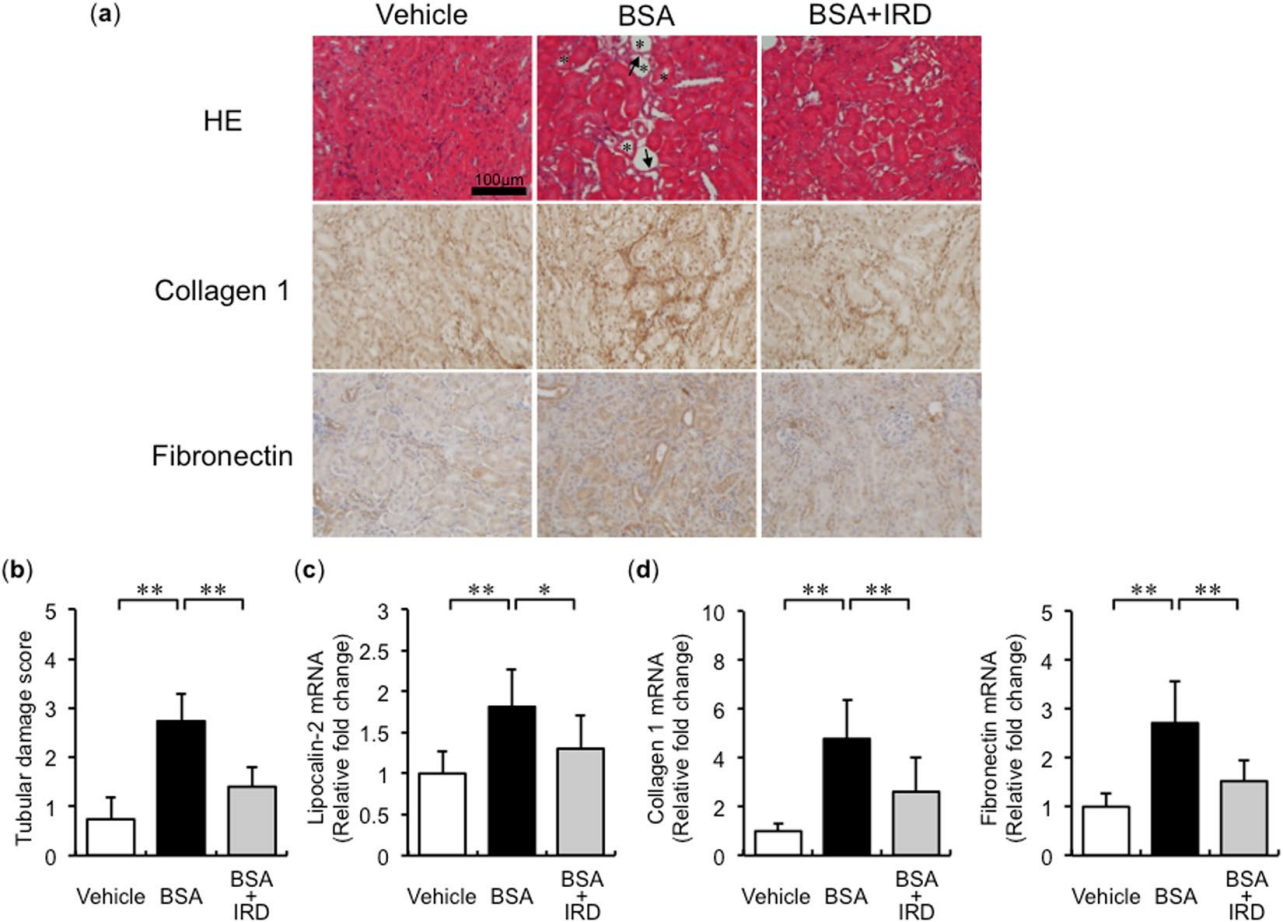
Dietary iron restriction inhibits BSA-induced tubulointerstitial injury. (**a**) Representative haematoxylin and eosin staining (HE), and immunohistochemical analysis of collagen 1 and fibronectin of the kidney section in the vehicle-injected and BSA-injected mice with normal diet (ND) or iron-restricted diet (IRD). (Arrow, atrophy and flattened tubule; Asterisk, tubular dilatation) (**b**) Quantitative analysis of interstitial damage scores. Values are expressed as mean ± SD. *P < 0.05, **P < 0.01; *n* = 8 in each group. The mRNA expression levels of (**c**) lipocalin-2, (**d**) collagen 1, and fibronectin in the kidneys of mice in each group. Values are expressed as mean ± SD. *P < 0.05, **P < 0.01, *n* = 6–9 in each group.

Renal tubulointerstitial injury was induced in mice with BSA overload, and it was alleviated by dietary iron restriction (Fig. 1a (upper) and b). Similar to histological analysis, the mRNA expression of lipocalin-2, a marker of tubulointerstitial damage, was also upregulated in BSA-overload mice, which was inhibited by iron-restricted diet (IRD, Fig. 1c). BSA-induced upregulation of fibrosis-related genes, such as collagen 1 and fibronectin, was also suppressed in mice with IRD (Fig. 1a (middle and lower), and d). These results suggested the favourable effect of dietary iron restriction in protein-overload tubulointerstitial injury.

### Dietary iron restriction attenuates the BSA-induced renal inflammatory changes

**Figure 2:**
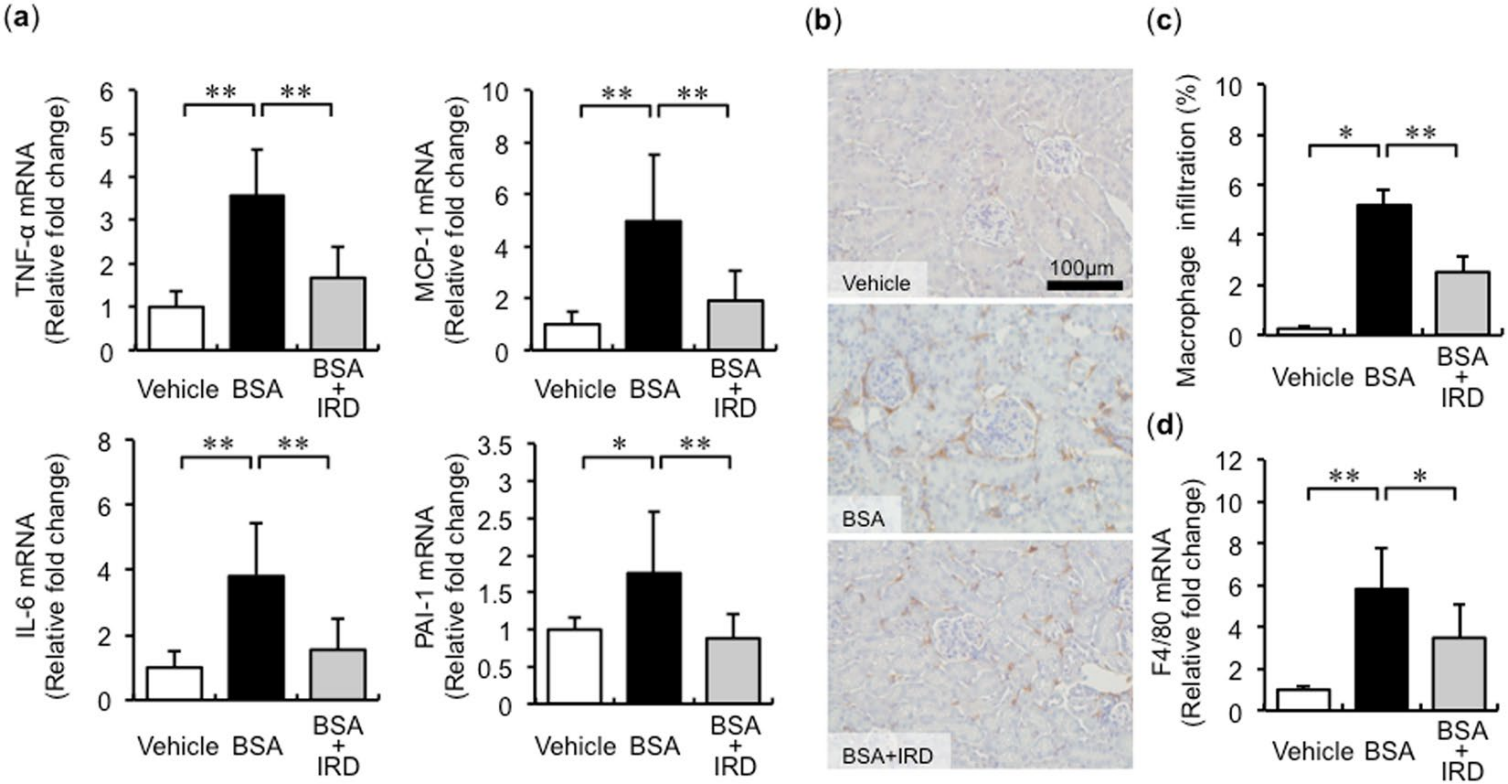
Iron restriction prevents BSA-induced inflammatory changes. (**a**) Quantitative analysis of mRNA expression for inflammation-related genes in the kidneys of mice in each group. Values are expressed as mean ± SD. *P < 0.05, **P < 0.01, *n* = 6–9 in each group. (**b**) Representative figures of F4/80 immunohistochemical staining in the kidney. (**c**) Semiquantitative analysis of the F4/80-positive spot in the kidneys of mice in each group. Values are expressed as mean ± SD. *P < 0.05, **P < 0.01, *n* = 8 in each group. (**d**) Quantification of F4/80 mRNA in the kidneys of mice in each group. Values are expressed as mean ± SD. *P < 0.05, **P < 0.01, *n* = 8 in each group.

BSA augmented renal inflammatory cytokines, including TNF-α, MCP-1, IL-6, and PAI-1, which were abolished by iron restriction (Fig. 2a). Increment of macrophage infiltration in the tubulointerstitium and increased F4/80 mRNA expression was observed in mice with BSA overload and was reduced by IRD (Fig. 2b–d).

### BSA-induced renal oxidative stress was reduced by iron restriction

**Figure 3:**
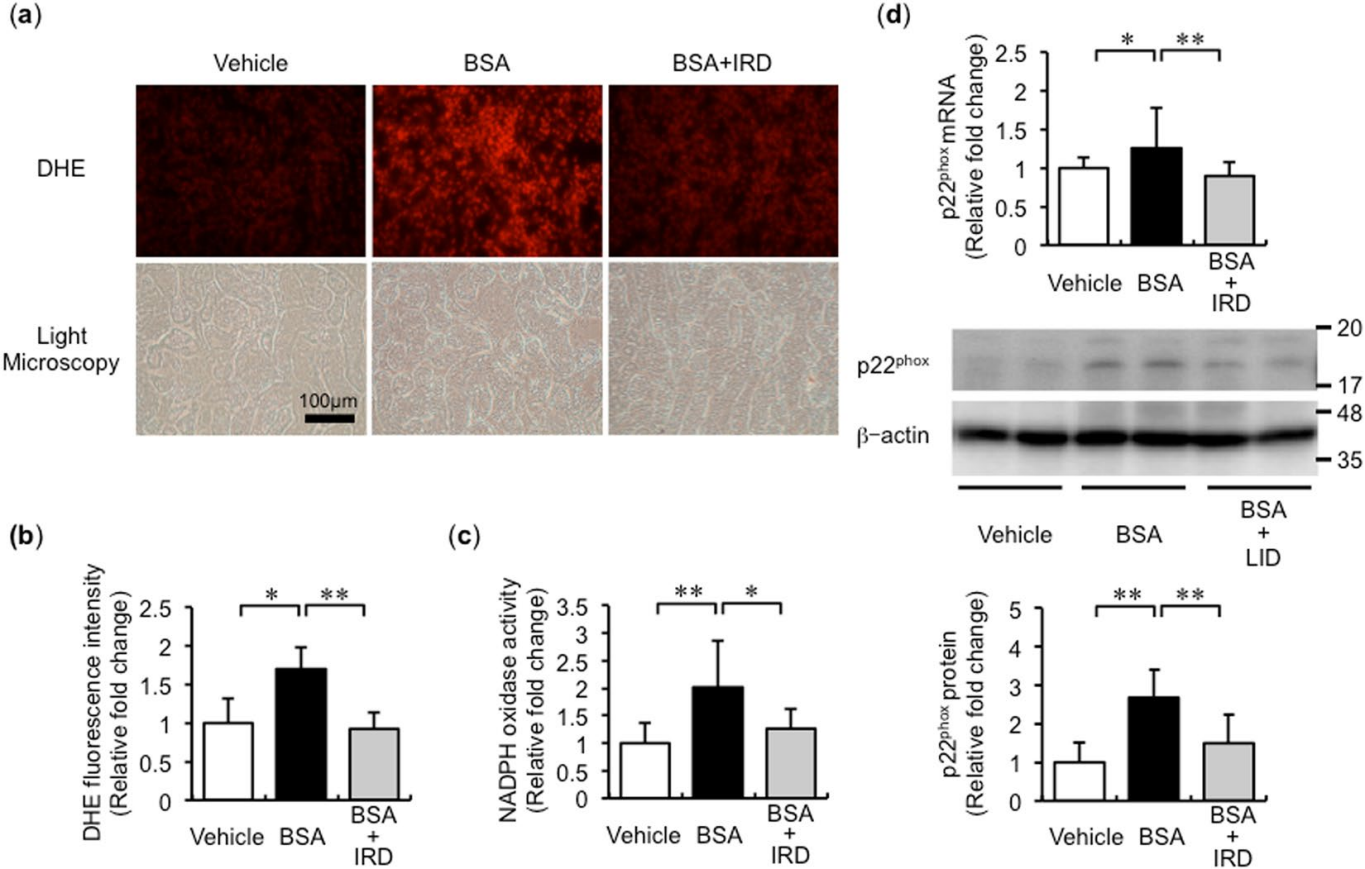
Inhibitory effect of iron restriction against BSA-induced oxidative stress in the kidney. (**a**) Representative images of dihydroethidium (DHE) staining in the kidneys of mice in each group. (**b**) Semiquantitative analysis of fluorescence intensity. Values are expressed as mean ± SD, *P < 0.05, **P < 0.01, *n* = 8 in each group. (**c**) NADPH activity in the kidney. Values are expressed as mean ± SD, *P < 0.05, **P < 0.01, *n* = 8 in each group. (**d**) p22^phox^ mRNA expression in the kidneys of mice in each group. Values are expressed as mean ± SD, *P < 0.05, **P < 0.01, *n* = 7–9 in each group. (**e**) p22^phox^ protein expression in the kidney. Upper panel, representative figures of p22^phox^ and β-actin from the kidney (full-length blots are presented as Supplementary Figure 1); and lower panel, semiquantitative analysis of densitometry for p22^phox^ expression. Values are expressed as mean ± SD, *P < 0.05, **P < 0.01, *n* = 10–12 in each group.

BSA promotes oxidative stress production through NADPH oxidase-dependent pathway^6^. We performed dihydroethidium (DHE) staining to assess oxidative stress production. BSA-induced renal oxidative stress in mice kidneys was reduced by IRD (Fig. 3a and b). The increase in NADPH oxidase activity was elevated in the BSA + ND group mice kidneys and was inhibited by IRD (Fig. 3c). Both mRNA and protein expression of p22^phox^, a subunit of the NADPH oxidase complex, was augmented in the BSA + ND group mice kidneys and was inhibited in the BSA + IRD group mice (Fig. 3d).

### NLRP3 inflammasome activation was abolished by dietary iron restriction

**Figure 4:**
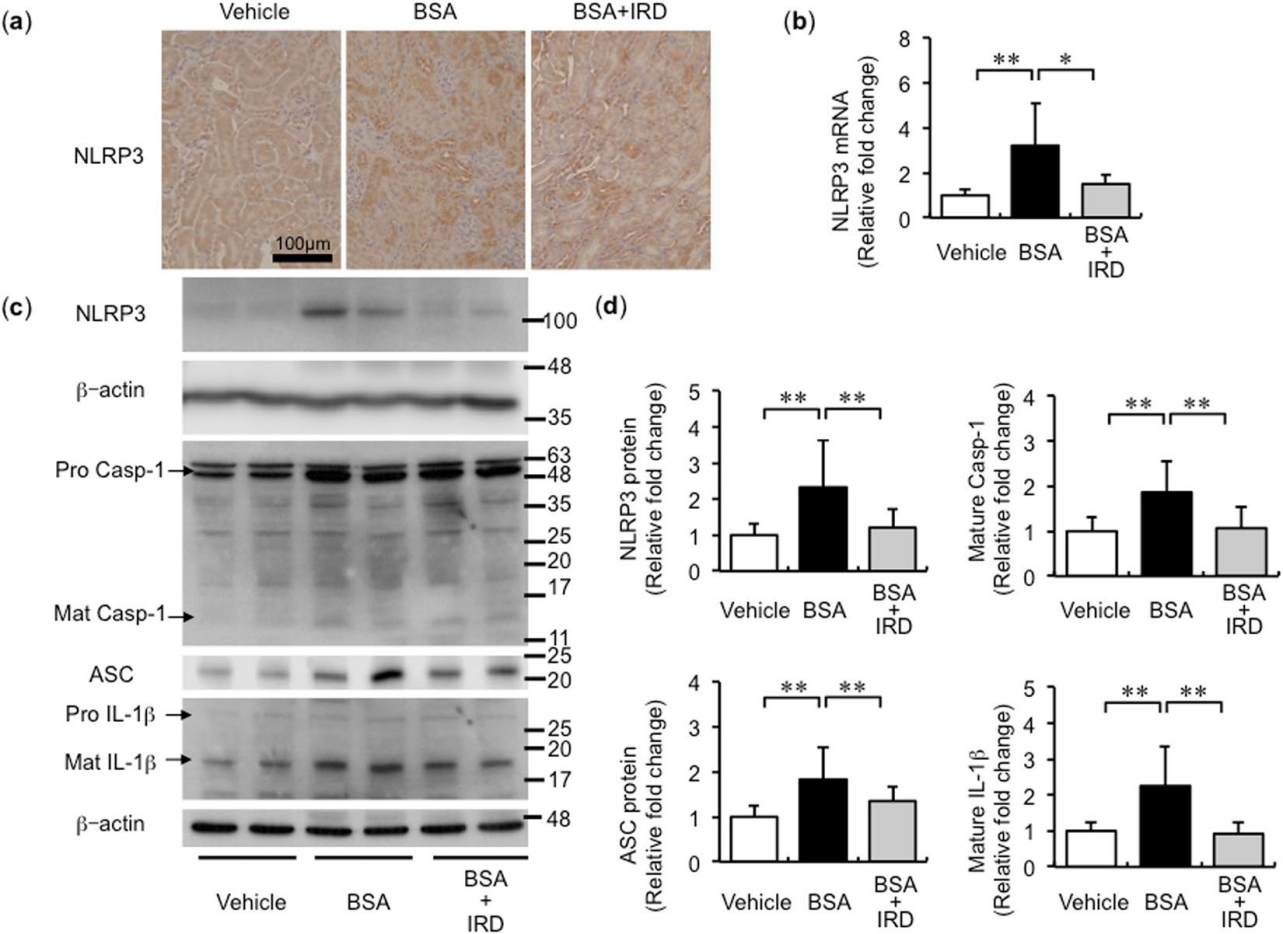
BSA-induced inflammasome activation inhibited in the kidneys of mice by dietary iron restriction. (**a**) Representative figures of nucleotide-binding oligomerisation domain-like receptor pyrin domain-containing protein 3 (NLRP3) staining in the kidneys of mice in each group. (**b**) NLRP3 mRNA expression in the kidneys of mice. Values are expressed as mean ± SD, *P < 0.05, **P < 0.01, *n* = 7–9 in each group. (**c**) Representative protein bands of NLRP3, caspase-1, ASC, IL-1β, and β-actin in the kidneys of mice (full-length blots are presented as Supplementary Figure 1). (**d**) Semiquantitative analysis of densitometry for NLRP3, mature caspase-1, ASC, and mature IL-1β in the kidney. Values are expressed as mean ± SD, *P < 0.05, **P < 0.01, *n* = 12 in each group.

BSA + ND group mice exhibited upregulation of mRNA and protein expression of NLRP3, predominantly in the renal tubule, and this increase was diminished by IRD (Fig. 4a–d). Similarly, the expression of p10 caspase-1, ASC, and mature IL-1β was also upregulated by BSA overload and was inhibited by IRD (Fig. 4c and d).

### Changes in iron content and ferrous iron in the kidney

**Figure 5:**
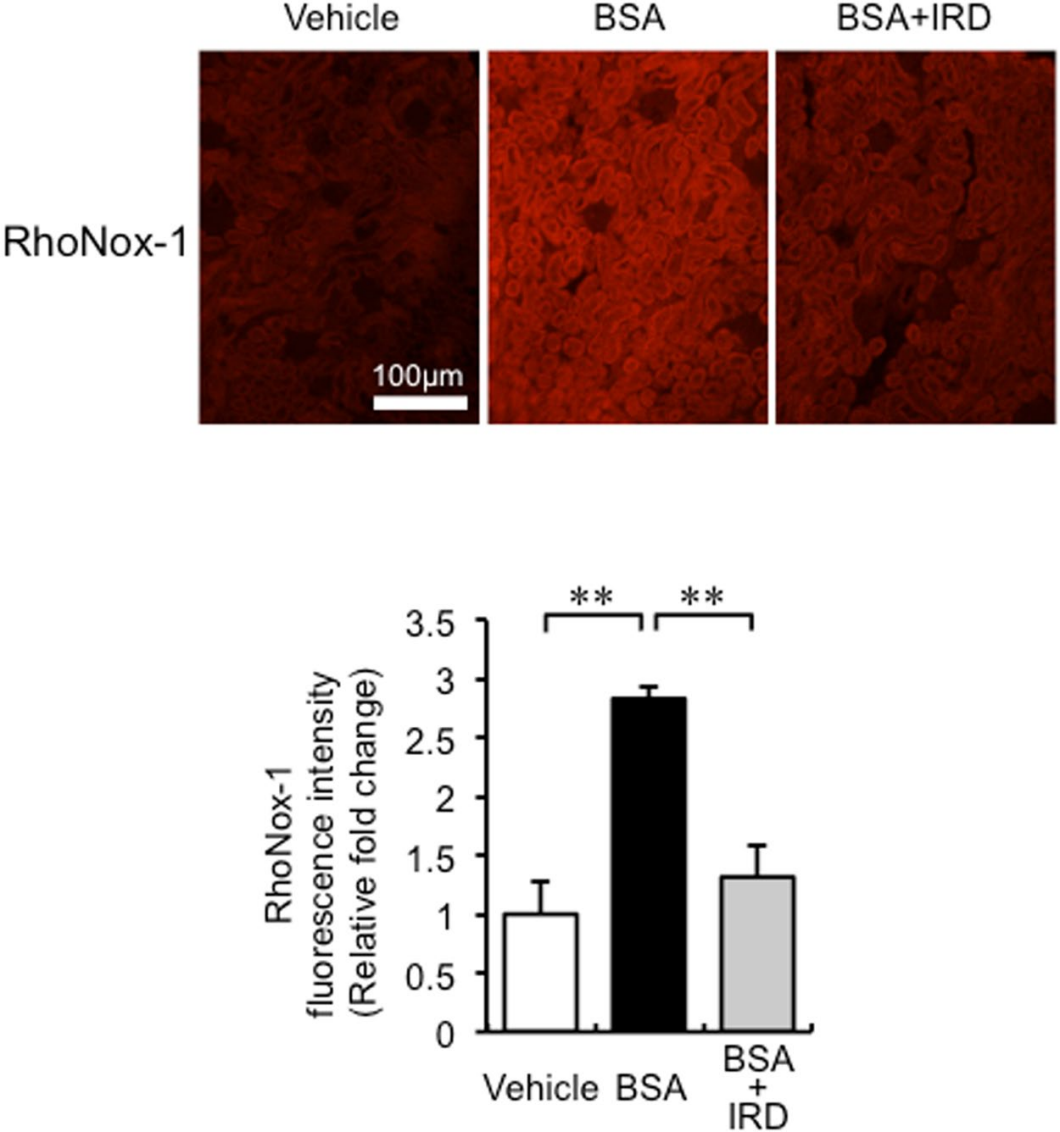
The effect of BSA overload on ferrous iron in the kidney. Upper panels, representative figures of RhoNox-1 staining in kidneys in each group; lower panel, semiquantitative analysis of RhoNox-1 staining of kidneys in each group. Values are expressed as mean ± SD, **P < 0.01, *n* = 4 in each group.

Dietary iron restriction reduced renal iron content and induced mild anaemia in the mice with BSA overload. Although BSA overload did not alter the total iron content in the kidney compared to the vehicle group, renal ferrous iron was increased by BSA overload, and the increase was suppressed by IRD (Table 1 and Fig. 5).

**Table 1:** Effects of bovine serum albumin (BSA) treatment and iron-restricted diet (IRD) on body weight, kidney weight, kidney iron content, red blood cell count, haemoglobin, haematocrit, mean corpuscular volume (MCV), mean corpuscular haemoglobin (MCH), and mean corpuscular haemoglobin concentration (MCHC) in mice.

	Vehicle	BSA	BSA + IRD
Body weight (g)	23.4 ± 1.1	23.7 ± 1.7	24 ± 2
Right kidney weight (mg)	109 ± 7	131 ± 18**	129 ± 10**
Right kidney weight to body weight ratio	4.6 ± 0.2	5.5 ± 0.5**	5.4 ± 0.2*
Renal iron content (µg/g tissue)	9.9 ± 3.6	9.7 ± 3	5.4 ± 1.9*^#^
Red blood cell (×10^4^/µL)	827 ± 63	829 ± 48	760 ± 49*^#^
Haemoglobin (g/dL)	12.6 ± 1	12.4 ± 0.7	10.6 ± 1.1**^##^
Haematocrit (%)	38.1 ± 3.3	38.1 ± 2	32.1 ± 3.1**^##^
MCV (fL)	46.1 ± 1	45.8 ± 1.1	42.4 ± 1.6**^##^
MCH (pg)	15.2 ± 3.3	15 ± 0.2	14 ± 0.6**^##^
MCHC (g/dL)	33 ± 0.7	32.6 ± 0.4	33.3 ± 0.4^#^

Data represent mean ± SD; *n* = 8 or 9; *P < 0.05, **P < 0.01 vs. control mice; ^#^ *P* < 0.05, ^##^ *P* < 0.01 vs. BSA-treated mice.

### Alteration of renal function and iron-related parameters in the mice with BSA overload

BSA overload resulted in an increase in the blood urea nitrogen (BUN) level, which was ameliorated by IRD. On the other hand, no change in the plasma creatinine level was observed between the three groups. Additionally, no change in the plasma ferritin level was observed between the mice administered with vehicle and the mice with BSA overload. Plasma iron and transferrin saturation (TSAT) were observed to be elevated in the BSA-treated mice. Plasma ferritin, plasma iron and TSAT levels were diminished in the BSA-treated mice with IRD. Plasma hepcidin-1 was also found to be elevated in the mice with BSA overload, which was dramatically reduced by IRD (Table 2).

**Table 2:** Effects of bovine serum albumin (BSA) treatment and iron-restricted diet (IRD) on the levels of plasma total protein, plasma albumin, blood urea nitrogen (BUN), plasma creatinine, plasma ferritin, plasma iron, transferrin saturation (TSAT), and plasma hepcidin-1 in mice.

	Vehicle	BSA	BSA + IRD
Plasma total protein (g/dL)	6.3 ± 1.2	9.2 ± 1.1**	9.2 ± 0.9**
Plasma albumin (g/dL)	2.8 ± 0.4	5.8 ± 0.9**	5.6 ± 1.1**
BUN (mg/dL)	29.2 ± 5.0	40.0 ± 9.8**	32.4 ± 5.7^#^
Plasma creatinine (mg/dL)	0.61 ± 0.08	0.61 ± 0.09	0.59 ± 0.09
Plasma ferritin (ng/mL)	814.2 ± 178.6	631.8 ± 170.0	323.2 ± 145.91**^##^
Plasma iron (µg/dL)	60.0 ± 7.3	78.2 ± 9.6**	61.9 ± 12.1^##^
TSAT (%)	25.1 ± 5.74	38.5 ± 6.8**	25.8 ± 8.3^##^
Plasma hepcidin-1 (ng/dL)	51.0 ± 33.8	112.6 ± 30.8**	0.4 ± 0.11*^##^

Data represent mean ± SD; *n* = 5–13; *P < 0.05, **P < 0.01 vs. control mice; ^#^ *P* < 0.05, ^##^ *P* < 0.01 vs. BSA-treated mice.

## Discussion

Proteinuria is an early biomarker for patients with CKD and a causal factor for the progression of CKD by eliciting tubular damage and tubulointerstitial injury. We first demonstrated that BSA overload enhanced ferrous iron levels in the kidney, although the renal total iron content was not altered. Renal iron content is increased in the mice with unilateral ureteral obstruction (UUO) surgery^17^ or in mice with 5/6 nephrectomy^16^, indicating that the increment in ferrous iron is due to the increase of total iron content. Furthermore, dietary iron reduction prevented the progression of diabetic nephropathy in the *db/db* mice, however, no difference was observed in the renal iron content of the control mice and that of the *db/db* mice^12^, suggesting the involvement of the labile ferrous iron, rather than the total iron content, in the pathological condition. In cisplatin-induced nephropathy and ischemia-reperfusion injury, bleomycin-detectable iron (iron capable of catalysing the reaction of free radicals is same as ferrous iron) and hydroxyl radical formation increased in the mice kidneys^18, 19^. It has been clinically observed that urinary catalytic iron increased during diabetic nephropathy or glomerulonephritis^20^. Thus, ferrous iron increased in these kidney diseases, and may be responsible for their pathological condition. Although the primary mechanism of ferrous iron augmentation remains unclear, labile ferrous iron is potentially involved in different types of kidney injury, and BSA overload-mediated increase in ferrous iron can also be a trigger for causing renal tubulointerstitial injury.

Therefore, protein overload-induced increment in renal ferrous iron suggested the promotion of oxidative stress *via* the Fenton reaction. As expected, renal superoxide and labile iron levels were reduced by IRD. Similar to this study, it has been observed that renal injuries induced by cisplatin or ischemia–reperfusion were diminished by iron chelation, through the inhibition of hydroxyl radical production, resulting in a decrease in the ferrous iron levels^18, 21^. In addition to the Fenton reaction, NADPH oxidase activity and p22^phox^ expression was upregulated in the kidneys of mice with protein overload, which was inhibited by dietary iron restriction. Protein overload augments NADPH oxidase activity^6^. We demonstrated that iron reduction diminished NADPH oxidase activity and p22^phox^ expression in diabetic nephropathy and renal fibrosis induced by unilateral ureteral obstruction^12, 17^. This supported the findings of our present study, since iron was essential for enzymatic activity of NADPH oxidase^22^ and biosynthesis of p22^phox^ subunit, which is a haemprotein^23^. Therefore, protein overload causes oxidative stress through the iron-dependent Fenton reaction and NADPH oxidase in the kidney, and these changes were inhibited by iron reduction, contributing to the alleviation of BSA-induced tubulointerstitial injury.

Consistent with our study, other studies have reported that both pharmacological and dietary iron reduction ameliorated the progression of CKD in various disease models^12–14, 16, 17^. In the present study, BSA-induced tubulointerstitial injury was diminished by iron restriction through the inhibition of oxidative stress, macrophage infiltration, inflammatory cytokines, and fibrosis-related genes. Oxidative stress is well-known to elicit an inflammatory response by activating transcription factors, such as NF-κβ^24^. Consistent with these findings, iron reduction has been known as an effective approach for reducing macrophage infiltration, inflammatory cytokines, and fibrosis in CKD such as diabetic nephropathy^12–14^, 5/6 nephrectomy^16^, and UUO-induced renal fibrosis^17^. This supports the inhibitory effect of iron restriction on renal tubulointerstitial injury induced by BSA overload by suppressing inflammation *via* reduced oxidative stress production.

The NLRP3 inflammasome plays a crucial role in regulating the caspase-1 activity and cleaving pro-IL-1β into its secreted form^25^. The NLRP3 inflammasome also contributes in promoting renal inflammation and CKD progression^26^ and implicates tubulointerstitial injury induced by protein overload^7, 8^. Similar to the above-mentioned studies, in the present study, BSA overload promoted NLRP3 inflammasome activation in the kidneys, which was suppressed by dietary iron restriction. Regarding the effect of iron on the inflammasome, cellular labile iron acts as a trigger for activating the NLRP3 inflammasome through iron-mediated reactive oxygen species (ROS) production in the macrophage^27^ and retina^28^. ROS is an upstream signalling molecule for NLRP3 inflammasome activation^29^. Collectively, the activation of NLRP3 inflammasome may be caused by ferrous iron-mediated oxidative stress production *via* the Fenton reaction or NADPH oxidase activation in the kidney. Therefore, iron restriction is also effective in inhibiting BSA-induced augmentation of renal ferrous iron, resulting in the suppression of NLRP3 inflammasome activation by reducing oxidative stress.

Among the biochemical parameters for determining iron status, the levels of plasma hepcidin-1 and TSAT were observed to be elevated in the mice with BSA overload. Hepcidin, a hormone secreted by hepatocytes, is an important regulator for iron metabolism. It acts by downregulating the expression of ferroportin, which is an iron exporter^30^. Patients with CKD display an elevated level of hepcidin^31^. Plasma hepcidin concentration, as well as mRNA levels of hepatic hepcidin, is also increased in adenine-induced CKD in mice^32^. Increased hepcidin level impairs the proper use for intracellular stored iron and the absorption of iron from the duodenum, resulting in the disturbance of systemic iron metabolism in CKD. Therefore, the increase in hepcidin level induced by BSA overload might also be involved in the alteration of iron homeostasis. Although TSAT was higher in BSA-treated mice, there was no difference of stored iron as indicated by plasma ferritin levels and renal iron concentration between vehicle-treated mice and BSA-treated mice, and plasma iron levels were elevated by BSA treatment, which disagree impaired utilization of iron regardless of hepcidin elevation. Further studies are needed to clarify the link between renal dysfunction and systemic iron homeostasis in CKD.

Patients with advanced CKD and end-stage renal dysfunction with renal anaemia are treated with the erythropoiesis-stimulating agent (ESA) along with iron, which is necessary for the effective functioning of ESA^33^. Further titration studies are necessary to clarify the optimal iron intake and iron status in CKD with respect to kidney function and hematopoiesis. In addition, iron deficiency is also known to stimulate fibroblast growth factor 23 (FGF23), a bone-derived phosphaturic hormone. The level of FGF23 is elevated in patients with CKD. Such elevated FGF23 levels are independently associated with the progression of CKD, cardiovascular events, and mortality^34^. Therefore, the beneficial effects of IRD on CKD might be offset by an increase in the level of FGF23. Further investigation is required to elucidate the beneficial and adverse effects of iron restriction on CKD.

In conclusion, augmentation of ferrous iron induced by protein overload causes oxidative stress *via* the Fenton reaction and NADPH oxidase, resulting in renal tubulointerstitial injury and inflammatory changes, which are ameliorated by iron restriction. These observations indicate that iron-mediated oxidative stress results in the pathogenesis of protein overload-induced renal injury.

## Materials and Methods

### Materials

We purchased and used the following commercially available antibodies: anti-interleukin-1β, anti-ASC (apoptosis-associated speck-like protein containing a CARD), anti-caspase-1 (p10), anti-fibronectin (Santa Cruz Biotechnology, Inc., Dallas, TX), anti-NLRP3 (Cell Signalling Technology, Danvers, MA), anti-F4/80 (Bio-Rad Laboratories, Hercules, CA), anti-type I collagen (SouthernBiotech, Birmingham, AL), and anti-β-actin (protein loading control, Cell Signalling Technology).

### Animal preparation and procedure

All experimental procedures for mice were performed in accordance with the guidelines of the Animal Research Committee of Tokushima University Graduate School, and the protocol was approved by the Institutional Review Board of Tokushima University Graduate School for animal protection (Permit Number: 14137). The mice were randomly divided into three groups: vehicle with normal diet (ND)-fed group, BSA with ND-fed group, and BSA with IRD-fed group. Seven-week-old male C57BL/6J mice were obtained from Nippon CLEA (Tokyo, Japan) and were maintained with *ad libitum* access to water and food. Then, mice were either fed with commercially available ND [D03072502 (3.9 mg Fe/100 g food)] or IRD [D03072501 (0.3 mg Fe/100 g food)] (Research Diets, Inc. New Brunswick, NJ). After 1 week, the mice were intraperitoneally injected with BSA. A stepwise increment dose regime of BSA was employed for inducing protein overload tubulointerstitial injury in the mice^7^. In brief, the initial BSA dose was 2 mg/g body weight on the first day and was increased gradually to the maximum dose of 10 mg/g body weight on day 5, which was maintained for 7 days. For the vehicle-treated group, the mice received intraperitoneal injection of normal saline. On day 12, 24 h after the last BSA dose, the mice were euthanised by intraperitoneally injecting an overdose of pentobarbital, and kidneys were removed and stored at −80 °C until further use.

### Preparation of BSA solution

BSA [Cohn fraction V (019–23293); Wako Pure Chemical Industries, Ltd., Osaka, Japan] was dissolved in normal saline, and the final concentration was 330 mg/mL. The concentration of endotoxin in BSA solution was 0.03 ng/mL, as detected by the limulus amebocyte lysate assay (Pierce™ LAL Chromogenic Endotoxin Quantitation Kit, Thermo Fisher Scientific, Inc., Waltham, MA).

### Real-time PCR for mRNA quantification

RNA extraction, cDNA synthesis, and quantitative RT-PCR methods have been described previously in detail^12^. In brief, the tissues were homogenised in the RNAiso reagent (TAKARA Bio, Inc., Otsu, Japan) using a Minilys tissue homogenizer (Bertin Instruments, Montigny-le-Bretonneux, France). RNA extraction and cDNA synthesis were performed according to the manufacturer’s instructions [PrimeScript RT reagent kit with gDNA Eraser (Perfect Real Time); TAKARA Bio, Inc.]. Quantitative RT-PCR was performed using the CFX Connect Real-Time PCR Detection System (BIO-RAD Laboratories Inc., Hercules, CA, USA) with THUNDERBIRD SYBR qPCR Mix (TOYOBO CO., LTD., Osaka, Japan). The primer sets used were as follows: 5′- ACGGCATGGATCTCAAAGAC-3′ and 5′-GTGGGTGAGGAGCACGTAGT-3′ for *TNF-α*, 5′-GGAGCTCATGATGTGAGCAA-3′ and 5′-GACCAGGCAAGGGAATTACA-3′ for monocyte chemotactic protein-1 (*MCP-1*), 5′- CCGGAGAGGAGACTTCACAG-3′ and 5′-TCCACGATTTCCCAGAGAAC-3′ for *IL-6*, 5′-GAGCGGAGAGTACTGGATCG-3′ and 5′-GTTCGGGCTGATGTACCAGT-3′ for *collagen-1*, 5′-GTCTTTCCGACCAAGAGCAG-3′ and 5′-GACAAAGGCTGTGGAGGAAG-3′ for *PAI-1*, 5′-ACAGAGCTCAACCTCCCTGA-3′ and 5′-TGTGCTCCTGGTTCTCCT-3′ for *fibronectin*, 5′-CTGTAACCGGATGGCAAACT-3′ and 5′-CTGTACCCACATGGCTGATG-3′ for *F4/80*, 5′- GTGGACTCCCATTGAGCCTA-3′ and 5′-CTCCTCTTCACCCTCACTCG-3′ for *p22* ^*phox*^, 5′-TGGAAGAACCAAGGAGCTGT-3′ and 5′-GGTGGGGACAGAGAAGATGA-3′ for *lipocalin-2*, and 5′-GCTCCAAGCAGATGCAGCA-3′ and 5′-CCGGATGTGAGGCAGCAG-3′ for *36B4* (internal control). The expression levels of all target genes were normalised using *36B4* expression, and the values were compared to the control group in terms of relative fold changes.

### Protein extraction and western blot analysis

Protein preparation and western blotting were performed as previously described^12^. In brief, kidney tissue samples were homogenised with the Minilys bead-based homogeniser (Bertin Instruments, Montigny-le-Bretonneux, France) and proteins were extracted. The extracted proteins were boiled for 5 min in the Laemmli sample buffer and separated using SDS–PAGE. Proteins were transferred onto a polyvinylidene fluoride (PVDF) membrane and the membrane was blocked for 1 h at room temperature. Next, the membrane was incubated individually with each primary antibody overnight at 4 °C, followed by incubation for 1 h with the secondary antibody. Then, immunoreactive bands were detected using a chemiluminescent reagent and visualised by exposure onto an X-ray film or by C-DiGit Blot Scanner (LI–COR, Lincoln, Nebraska, USA). Densitometry of the visualised bands was quantified using the Image J 1.38x software (https://imagej.nih.gov/ij/).

### Histological analysis for tubulointerstitial damage

The assessment of renal tubulointerstitial damage has been described previously^35^. In brief, the kidney tissue samples were fixed in 4% paraformaldehyde and embedded in paraffin. Samples were cut into 3-µm sections, and the sections were stained with haematoxylin and eosin (HE). Tubular injuries were scored in a blinded manner according to the percentage of damage (including atrophy, flattening of the proximal tubule epithelial cells, and tubular dilation) as follows: 0, normal; 1, <20%; 2, 20–40%; 3, 40–60%; 4, 60–80%, and 5, >80%. Ten random microscopic fields per kidney section were used for quantification.

### Immunohistochemistry of the kidney samples

Immunohistochemical staining of F4/80 and fibronectin was performed as described previously^17^. In brief, the paraffin-embedded kidney samples were cut into 3-µm sections, deparaffinised, processed for antigen retrieval using 10 mM citrate buffer at 95 °C for 20 min, and cooled for 20 min. For collagen-1 staining, the frozen tissue sections were used^36^. Non-fixed kidney tissues were embedded in the Tissue-Tek O.C.T. Compound (Sakura Finetek, Tokyo, Japan) and subjected to snap freezing using the liquid nitrogen–cold isopentane mixture. Samples were cut into 8-µm sections, dried, and fixed in 10% neutral formaldehyde for 10 min. After blocking, the sections were incubated with the primary antibody at 4 °C overnight. Antibody distribution was visualised using a streptavidin–biotin complex assay and a 3,3′-diaminobenzidine (DAB) substrate kit (LSAB + Kit Universal; Dako Japan, Tokyo, Japan). Sections incubated without the primary antibody were used as negative controls. The evaluation of macrophage infiltration in tubulointerstitium was performed as described previously^17^. Briefly, ten microscopic fields were randomly selected in the renal cortex and the macrophage-positive area was expressed as a percentage of the total area, except the areas under tubular lumen, glomeruli, and vessels, using the ImageJ 1.38x software.

### Measurement of tissue iron content

Tissue iron content was measured using an iron assay kit according to the manufacturer’s instructions (Metallo assay; Metallogenics Co., Ltd., Chiba, Japan), as described previously^12, 17^. In brief, the kidney tissue samples were homogenised in the cell lysis buffer, and the non-centrifuged crude lysates with 0.05 M hydrochloric acid were mixed at regular intervals for 30 min, and centrifuged with 12,000 rpm for 10 min at 4 °C. Subsequently, the supernatants were used for assay. Tissue iron concentration was corrected using tissue weight and expressed as µg/g of wet tissue.

### Measurement of NADPH oxidase activity

NADPH oxidase activity was measured as previously described^12, 17^. In brief, the kidney tissue sample was immediately homogenised in the NADPH oxidase lysis buffer and sonicated for 3 s. The NADPH substrate (final concentration, 300 µM) was added to the renal suspension with lucigenin (5 µM). Luminescence was measured every second for 60 min in a plate reader (SpectraMax Paradigm FilterMaxF3; Molecular Devices Japan, Tokyo, Japan). NADPH activity was expressed as relative luminescence units normalised to the protein concentration.

### *In situ* oxidative stress detection in the kidney tissue

Detection of superoxide production in the kidney was evaluated by DHE staining method as previously described^12^. Non-fixed frozen tissue sections were washed with PBS, incubated with DHE in PBS (10 µM) in a dark, humidified container at room temperature for 30 min, and observed using fluorescence microscopy.

### Detection of renal ferrous iron

RhoNox-1 was used to detect ferrous iron content in the kidney^37^. The staining method used has been described previously^36^. First, frozen sections were washed three times in Hank’s balanced salt solution (HBSS) for 5 min, fixed in 10% neutral formaldehyde for 1 min, and washed three times with HBSS for 5 min. Next, the fixed sections were incubated with RhoNox-1 in HBSS (5 µM) in a dark, humidified container at room temperature for 30 min. After washing three times with HBSS, the sections were covered with a small drop of the mounting medium and observed using fluorescence microscopy. The section stained with RhoNox-1 was observed and quantified using fluorescence microscopy.

### Haematological and biochemical analysis

Peripheral blood samples were analysed at Shikoku Chuken, K.K. (Kagawa, Japan). The plasma total protein concentration was measured by the bicinchoninic acid assay (BCA) assay. The analysis of plasma albumin, BUN, and TSAT was performed at the Nagahama Life Science Laboratory (Shiga, Japan). The plasma creatinine level was measured using the LabAssay Creatinine kit (Wako Pure Chemical Industries, Ltd., Osaka, Japan). The plasma ferritin concentration was determined using the Mouse Ferritin ELISA kit (Immunology Consultants Laboratory, Newberg, OR) according to the manufacturer’s instructions. The hepcidin-1 concentration in mice was measured using surface-enhanced laser desorption/ionization time-of-flight mass spectrometry (SELDI-TOF–MS^38^; Medical Care Proteomics Biotechnology Co., Ltd., Kanazawa, Japan).

### Statistical analysis

Data are presented as mean ± standard deviation (SD). Significant differences between the three groups were determined using one-way ANOVA followed by Tukey’s post-hoc test. The differences between data were considered to be statistically significant at P < 0.05.

## Electronic supplementary material

Supplementary figure 1 and 2
