# Supplementary material for: Dietary iron restriction alleviates renal tubulointerstitial injury induced by protein overload in mice
Source: Sci Rep. 2017 Sep 6;7:10621. doi: 10.1038/s41598-017-11089-0 (PMC5587788; doi:10.1038/s41598-017-11089-0)

Dietary iron restriction alleviates renal tubulointerstitial injury induced by protein overload in mice

Yasumasa Ikeda[1*], Yuya Horinouchi[1], Hirofumi Hamano[1,2], Tasuku Hirayama[3], Seiji Kishi[4], Yuki Izawa-Ishizawa[1], Masaki Imanishi[2], Yoshito Zamami[2,5], Kenshi Takechi[6], Licht Miyamoto[7], Keisuke Ishizawa[2,5], Ken-ichi Aihara[8], Hideko Nagasawa[3], Koichiro Tsuchiya[7] & Toshiaki Tamaki[1]

[1]Department of Pharmacology, Institute of Biomedical Sciences, Tokushima University Graduate School, Tokushima, Japan

[2]Department of Pharmacy, Tokushima University Hospital, Tokushima, Japan

[3]Laboratory of Pharmaceutical and Medicinal Chemistry, Gifu Pharmaceutical University, Gifu, Japan

[4]Department of Nephrology, Institute of Biomedical Sciences, Tokushima University Graduate School, Tokushima, Japan

[5]Department of Clinical Pharmacology and Therapeutics, Institute of Biomedical Sciences, Tokushima University Graduate School, Tokushima, Japan

[6]Clinical Trial Center for Developmental Therapeutics, Tokushima University Hospital, Tokushima, Japan

[7]Department of Medical Pharmacology, Institute of Biomedical Sciences, Tokushima University Graduate School, Tokushima, Japan

[8]Department of Community Medicine for Diabetes and Metabolic Disorders, Institute of Biomedical Sciences, Tokushima University Graduate School, Tokushima, Japan

Supplementary Figure 1: Full-length blots for use as representative figures in the manuscript.

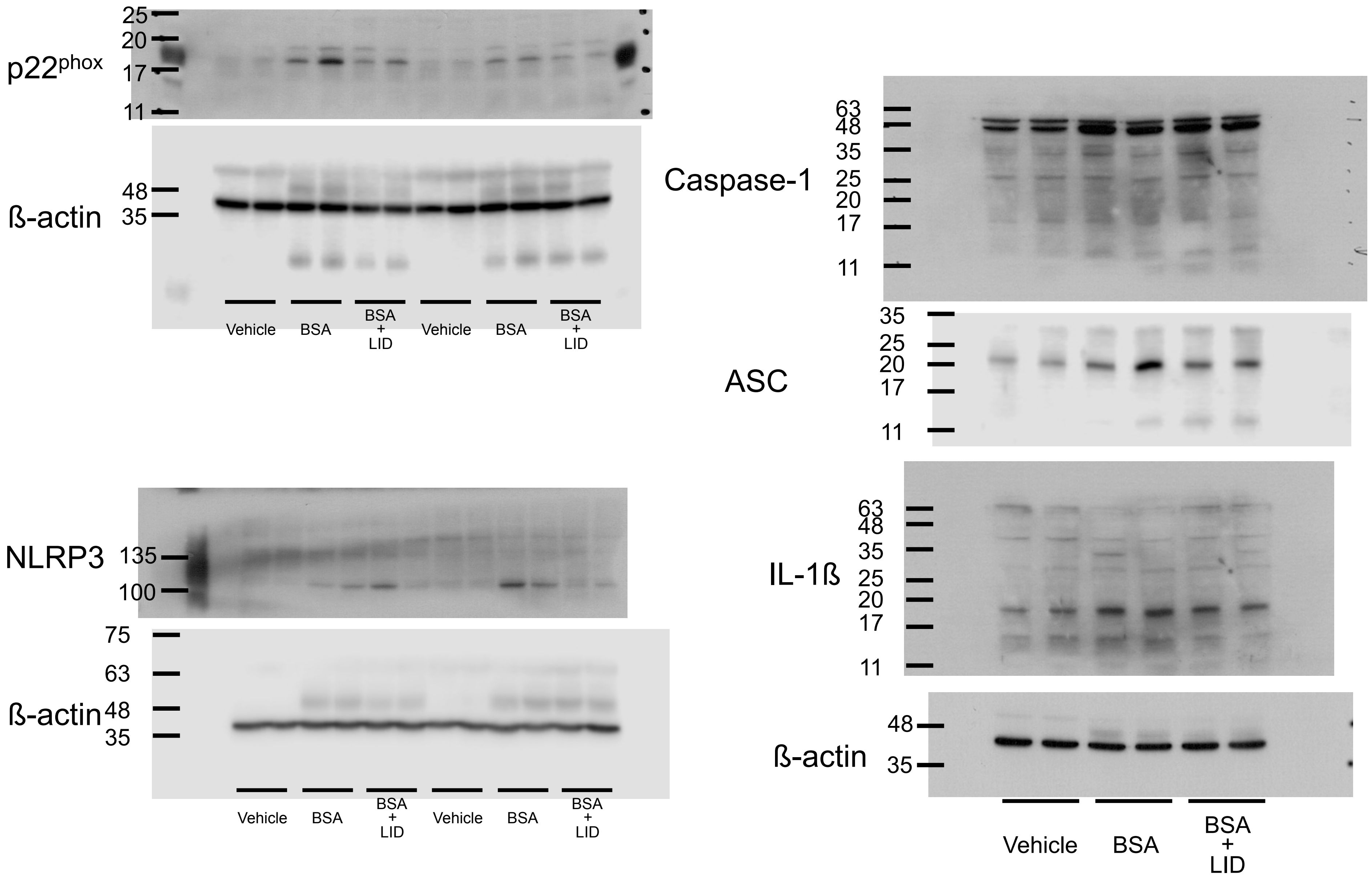

Supplementary Figure 2: Representative histological images in kidney stained with Masson's Trichrome.

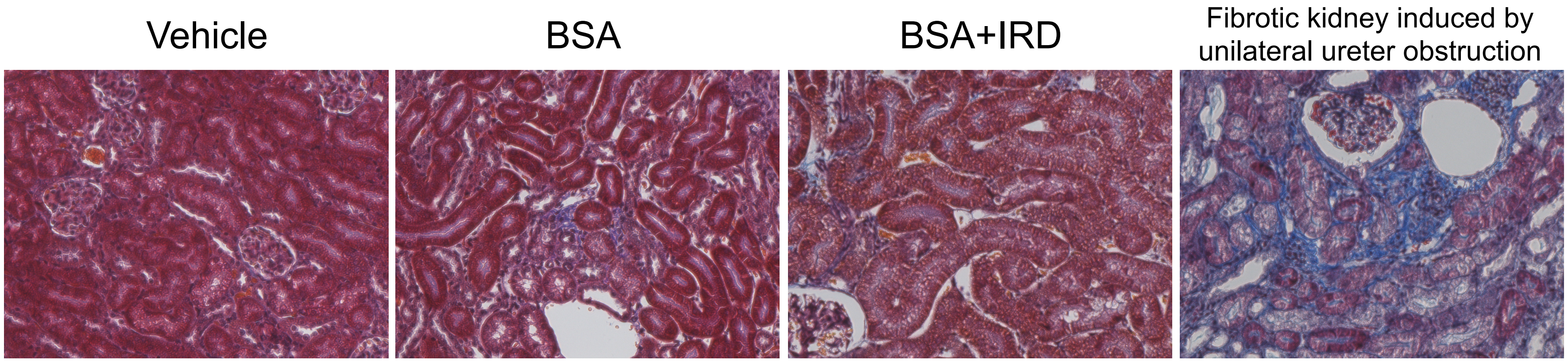

Supplement: Supplementary file 1 — Supplementary figure 1 and 2 [file 41598_2017_11089_MOESM1_ESM.pdf]
